# Effects of replacing wheat bran with palm kernel cake or fermented palm kernel cake on the growth performance, intestinal microbiota and intestinal health of tilapia (GIFT, *Oreochromis niloticus*)

**DOI:** 10.3389/fnut.2024.1368251

**Published:** 2024-02-21

**Authors:** Huajing Huang, Xiangqin Lin, Xiaoxue Meng, Yu Liu, Jiongting Fan, Lulu Zhu, Jiaxian Chen, Lu Zhang, Haifeng Mi, Junming Deng

**Affiliations:** ^1^College of Fisheries, Guangdong Ocean University, Zhanjiang, Guangdong, China; ^2^Aquatic Animals Precision Nutrition and High-Efficiency Feed Engineering Research Centre of Guangdong Province, Guangdong Ocean University, Zhanjiang, Guangdong, China; ^3^Tongwei Agricultural Development Co., Ltd., Chengdu, Sichuan, China

**Keywords:** palm kernel cake, fermented palm kernel cake, growth performance, intestinal microbiota, intestinal health

## Abstract

A nine-week feeding trial was conducted to evaluate the effects of replacing wheat bran (WB) with palm kernel cake (PKC) or fermented palm kernel cake (FPKC) on the growth performance, intestinal microbiota and intestinal health of genetically improved farmed tilapia (GIFT, *Oreochromis niloticus*) (initial weight 7.00 ± 0.01 g). Eleven isonitrogenous and isolipidic experimental diets were formulated by replacing 0, 20, 40, 60, 80, and 100% of dietary WB with PKC or FPKC. Replacement of WB with PKC concentrations up to 80% had no significant effect on the growth rate of tilapia or feed utilisation (*p* > 0.05). FPKC improved the growth performance of tilapia, with optimum growth achieved at 40% replacement level (*p* < 0.05). Complete replacement with PKC significantly decreased the activity of lipase and trypsin, and reduced the height of muscularis and the height of villus (*p* < 0.05). However, FPKC significantly increased amylase activity and villus height (*p* < 0.05). The apparent digestibility of dry matter and energy decreased linearly with increasing levels of PKC substitution, while FPKC showed the opposite trend (*p* < 0.05). PKC replacement of WB by 20% significantly reduced serum diamine oxidase activity and endothelin levels and increased intestinal tight junctions (*p* < 0.05). However, FPKC significantly decreased diamine oxidase activity and increased intestinal tight junctions (*p* < 0.05). PKC completely replaced WB, up-regulating the expression of pro-inflammatory factors (*il-1β*) (*p* < 0.05). When 40% of WB was replaced with FPKC, the expression of pro-inflammatory factors (*il-1β* and *il-6*) was decreased significantly (*p* < 0.05). Completely replacement of WB with PKC reduced the abundance of Firmicutes and Chloroflexi, while FPKC reduced the abundance of Fusobacteriota and increased the levels of Actinobacteriota. WB can be replaced with PKC up to 80% in tilapia feeds. However, the high percentage of gluten induced intestinal inflammation, impaired gut health, and reduced dietary nutrient utilisation and growth performance. Complete replacement of WB with FPKC promoted intestinal immunity. It also improved dietary nutrient utilisation and growth performance. However, the optimal growth was achieved at a 40% replacement level.

## 1 Introduction

The rapid development of China’s farming industry has led to an increasing demand for feed grain. In 2022, the amount of feed grain used in China’s farming industry was 299.3 million tons, accounting for 65.9% of the total feed consumption. It is projected that by 2030, the total demand for feed grain in China’s farming industry will reach 331.03 million tons (1). However, China’s limited land resources and the pessimistic prospects for increasing domestic food production have led to a heavy dependence on imported grains from various countries (2). Food competition between humans and farmed animals is a growing problem in China. In particular, there is fierce competition for wheat and the by-products of wheat processing (wheat bran [WB] and Wheat flour), which are commonly used as animal feed ingredients but are also popular foods for humans. Therefore, to protect national food security, there has been intense interest in the development of non-grain protein ingredients to replace WB in animal feed (3, 4). WB is a by-product of wheat processing and is widely used as a feed ingredient. However, the price of WB has been increasing in recent years, which has become an important factor restricting its application (3).

Among the many non-grain proteins available, palm kernel cake (PKC) has attracted significant interest for used as an aquatic feed due to its large-scale production and low price, as well as nutrient composition (5–7). PKC is mainly produced in Southeast Asia and African countries as a by-product of de-husking palm kernel for oil extraction (8). The global production of PKC is more than 10 million tonnes per year and is growing at a rate of about 10% (9). PKC has become the fifth largest protein meal commodity in the world, after soybean meal, rapeseed meal, sunflower meal, and cottonseed meal, and has a high feeding value (10). It is comparable to WB in crude protein content, which constitutes about 14.5% to 19.6% (5, 11, 12). It is currently used to supplement ruminant and livestock feeds (13–16). Existing studies have shown that PKC can be used to supplement feeds for Nile tilapia (*Oreochromis niloticus*) (17), red hybrid tilapia (*Oreochromis sp*) (18) and asian-african catfish (*Clarias macrocephalus* × *C. gariepinus*) (7) up to 20%. However, its supplementation in aquatic animal feeds is limited by its low crude protein content and high levels (approximately 42%) of non-starch polysaccharides (NSPS) (19, 20). However, NSPs adhere to the digestive tract, thereby hindering nutrient absorption (21). The amino acid imbalance in PKC, with very low levels of methionine, lysine, histidine, and threonine, are another limitation (22, 23). Currently, it has been proven by numerous scholars that balancing the amino acid profile in animal feed is essential for animal growth (24–27).

Currently, enzymatic hydrolysis and microbial fermentation are routinely used to promote PKC (8, 18, 28). However, we found that PKC can only be added to aquatic animals up to about 20% even after enzymatic hydrolysis or microbial fermentation (8, 18). At present, studies have shown that the effect of bacterium enzyme synergistic fermented of feed ingredients is better than the effect of bacterial or enzyme treatment alone, and the former shows obvious advantages in improving the nutritional value of feeds and feeding effect (29, 30). The synergistic effect of combining bacterium enzyme synergistic fermented overcomes the shortcomings of pure fermentation or enzymatic digestion of feed. The co-fermentation not only degrades the macromolecular constituents completely, but also generates useful metabolites, degrades anti-nutritional factors, improves the flavour and palatability of feeds, promotes animal feed intake, and increases the nutritive value of feeds and their utilisation (31). Currently, bacterial enzyme co-fermentation is widely used in a processing variety of raw materials (32–34). However, studies related to bacterium enzyme synergistic fermented involving PKC have yet to be reported.

The Genetically Improved Farmed Tilapia (GIFT) project has boosted the fish growth rate, increased the feed utilisation rate, and yielded economic benefits worldwide (35, 36). A large number of studies have analysed the nutritional value of the GIFT strain of tilapia. However, the effect of PKC or fermented palm kernel cake (FPKC) replacement of WB has yet to be reported. If PKC and FPKC can be reasonably developed and utilized to replace WB, it is of great significance to reduce China’s dependence on imported food, maintain national food security, alleviate the shortage of feed resources, and solve the contradiction between human and animal competition for food, and promote the sustainable development of aquaculture. Therefore, this study was conducted to comprehensively evaluate the effects of PKC or FPKC replacement WB on growth performance, intestinal microbiota, and intestinal health of tilapia, and to elucidate the effect of fermentation on PKC. The results provide a theoretical basis for enhanced utilisation of PKC and FPKC in aquatic animal feeds.

## 2 Materials and methods

### 2.1 Experimental diets

PKC and FPKC were provided by Tongwei Agricultural Development Co., Ltd. (Tongwei Co., Ltd., Chengdu, China). The fermentation process and the preparation of the bacterial and enzyme solutions were carried out strictly according to the methods provided by Vland Biotech (Qingdao, China). Details of the fermentation process are given in the Supplementary Material. The nutrient levels of PKC and FPKC are presented in Table 1.

**Table 1:** Nutritional composition of PKC and FPKC (%, dry matter basis).

Item	PKC	FPKC
Crude protein	17.58	18.50
Crude lipid	6.10	4.67
Aspartic acid	1.26	1.36
Threonine	0.49	0.66
Serine	0.63	0.78
Glutamic acid	3.05	3.06
Glycine	0.73	0.84
Alanine	0.73	1.24
Cystine	0.26	0.24
Valine	0.77	0.78
Methionine	0.24	0.24
Isoleucine	0.52	0.60
Leucine	1.07	1.26
Tyrosine	0.31	0.38
Phenylalanine	0.66	0.78
Lysine	0.38	0.58
Histidine	0.18	0.36
Arginine	1.79	1.16
Proline	0.45	0.90
Total amino acid	13.52	15.22

Tryptophan was not detected due to acid hydrolysis.

The experimental diets were based on fish meal, chicken meal, and soybean meal as protein sources and soybean lecithin and soybean oil as fat sources. Referring to the study of Li et al. (37), PKC and FPKC were used to replace 0%, 20%, 40%, 60%, 80%, and 100% of WB in the basal diets, respectively. A total of 11 isonitrogenous and isolipidic diets (30% crude protein and 7% crude lipid) were formulated (Table 2). The ingredients other than fats and oils were crushed and sieved through a 40-mesh sieve, and the ingredients were accurately weighed and thoroughly mixed according to the formula. Soybean lecithin and soybean oil were then added, stirred, and mixed well again, and finally, the appropriate amount of water was added, and pellets with a diameter of 2.50 mm were obtained by a double-screw extruder (F-26 type; South China University of Technology, Guangzhou, China). The feed was dried in an air-conditioned room for 48 h until the moisture content was about 10% and then stored in a refrigerator at −20°C for reserve. The corresponding molecular weight distributions of the proteins are shown in Supplementary Figure S1.

**Table 2:** Ingredients and proximate composition of the experimental diets (%, dry matter basis).

Item	Control	Replacement level of WB with PKC					Replacement level of WB with FPKC				
		20%	40%	60%	80%	100%	20%	40%	60%	80%	100%
Fish meal	3.00	3.00	3.00	3.00	3.00	3.00	3.00	3.00	3.00	3.00	3.00
Chicken meal	1.00	1.00	1.00	1.00	1.00	1.00	1.00	1.00	1.00	1.00	1.00
Rapeseed meal	10.00	10.00	10.00	10.00	10.00	10.00	10.00	10.00	10.00	10.00	10.00
Soybean meal	25.00	25.00	25.00	25.00	25.00	25.00	25.00	25.00	25.00	25.00	25.00
Palm kernel cake	0.00	6.00	12.00	18.00	24.00	30.00	–	–	–	–	–
Fermented palm kernel cake	–	–	–	–	–	–	6.00	12.00	18.00	24.00	30.00
Wheat bran	28.75	23.00	17.25	11.50	5.75	0.00	23.00	17.25	11.50	5.75	0.00
Wheat flour	22.99	22.99	22.99	23.00	23.02	23.01	22.90	22.81	22.72	22.65	22.56
Soybean oil	4.78	4.51	4.23	3.95	3.67	3.40	4.60	4.41	4.23	4.04	3.85
Soybean lecithin	0.50	0.50	0.50	0.50	0.50	0.50	0.50	0.50	0.50	0.50	0.50
L-lysine	0.20	0.22	0.25	0.27	0.29	0.32	0.22	0.25	0.27	0.29	0.32
DL-methionine	0.20	0.20	0.20	0.20	0.19	0.19	0.20	0.20	0.20	0.19	0.19
Additive premix^a^	1.20	1.20	1.20	1.20	1.20	1.20	1.20	1.20	1.20	1.20	1.20
Others^b^	2.38	2.38	2.38	2.38	2.38	2.38	2.38	2.38	2.38	2.38	2.38
*Proximate composition*											
Dry matter (DM, %)	90.51	90.29	90.55	90.52	90.54	90.92	90.34	90.93	89.69	89.76	90.08
Crude protein (% DM)	30.04	30.34	30.39	30.05	30.27	30.04	30.33	30.36	30.18	30.44	30.28
Crude lipid (% DM)	7.52	7.56	7.59	7.40	7.48	7.42	7.47	7.55	7.33	7.31	7.37
Ash (% DM)	7.06	7.05	7.07	6.96	7.06	6.87	7.18	7.32	7.40	7.55	7.81
Gross energy (KJ/g DM)	21.73	21.75	21.70	21.73	21.63	21.59	21.72	21.52	21.74	21.69	21.44

a Additive premix (g/kg mixture): vitamin A, 0.20 g; vitamin D_3_, 0.003 g; vitamin E, 4.40 g; vitamin K_3_, 0.66 g; vitamin B_1_, 0.33 g; vitamin B_2_, 0.88 g; vitamin B6, 0.73 g; vitamin B_12_, 0.001 g; nicotinic acid, 2.89 g; calcium pantothenate, 1.64 g; folic acid, 0.07 g; biotin, 0.003 g; vitamin C, 10.01 g; FeSO_4_·7H_2_O, 52.87 g; H_3_ClCu_2_O_3_, 0.65 g; ZnSO_4_·7H_2_O, 43.15 g; MnSO_4_·7H_2_O, 31.56 g; MgSO_4_·H_2_O, 44.65 g; Ca(IO_3_)_2_, 0.42 g; Na_2_SeO_3_, 0.11 g; CoCl_2_·6H_2_O, 0.14 g.

b Others included 1.80% Ca(H_2_PO_4_)_2_, 0.20% NaCl, 0.30% choline chloride, 0.03% vitamin C, 0.05% Y_2_O_3_.

### 2.2 Fish and experimental procedure

The experimental fish were purchased from Lianjiang branch of Hainan Baolu Aquatic Technology Co., Ltd. (Guangdong, China) and were temporarily reared for a fortnight, during which time they were fed commercial diets (Jiakang feed, Xiameng, China) to acclimatize them to the experimental conditions. Then 1,155 healthy, uniformly sized (7.00 ± 0.01 g) tilapia were randomly divided into 11 groups (3 replicates per group, 35 fish per net cage (1.2 m × 0.8 m × 1.0 m)). All cages were set up outdoors in two neighboring concrete tanks (5.8 m × 5.4 m × 2.1 m). During the culture period, the water quality was maintained at a natural temperature of 25–31°C, with dissolved oxygen >6.0 mg/L and ammonia nitrogen <0.02 mg/L. The culture experiment lasted for 9 weeks, and the fish were fed twice a day (08,00 and 17,00) until apparent satiation (most of the fish were not grabbing for food), and the amount of feed fed was recorded.

### 2.3 Sample collection

Digestibility tests were conducted during feeding trials according to the method described by Liu et al. (38). Y_2_O_3_ (99.9%, Sinopharm Chemical Reagent Co., Ltd., Shanghai, China) was used as an indicator for the experimental feed. The fecal collection was started 2 weeks after the fish were acclimatized to the experimental feed. Feces were collected daily from the bottom of the cages using a 100-mesh fishing net 2–5 h after feeding the fish. Intact feces were then selected and placed in the corresponding centrifuge tubes and finally dried at 65°C for 6 h and stored at −20°C for use. The fecal collection was continued for 7 weeks to ensure that fecal samples met the requirements for testing. At the end of the feeding trial, all fish were fasted for one day. Fish were then sampled after anesthetizing with eugenol (1:12000) (Shanghai Reagent Corporation, Shanghai, China). The number and total weight of fish in each net cage were recorded for the calculation of growth-related indices. Blood was collected from three randomly selected fish from each net cage using a 1 mL syringe, and the blood was injected into a 2.0 mL centrifuge tube, placed in a refrigerator at 4°C for 12 h, and centrifuged at 3500 r/min for 10 min at 4°C. The upper layer of serum was taken and stored in the refrigerator at −80°C. Four fish hindguts were randomly taken from each net cage and fixed in a 4% paraformaldehyde solution for subsequent histological analysis. Two fish foreguts and hindguts were randomly collected from each net cage and placed in enzyme-free blast tubes, which were quickly placed in liquid nitrogen and then transferred to a − 80°C refrigerator for storage for digestive enzyme activity and intestinal microbiota analysis. The hindgut of 3 fish per net cage was randomly collected and placed in RNA later solution and stored in a refrigerator at −80°C for relevant mRNA expression analysis.

### 2.4 Chemical composition analysis

The chemical compositions of the feed ingredients, experimental diets, and feces were determined with reference to the AOAC standard methods (39). The samples were dried in an oven at 105°C until constant weight, and the dry matter contents of the samples were measured. The crude lipid contents of the samples were determined by the Soxhlet method. The crude protein contents of the samples were determined by the Kjeldahl method. The gross energy of the feeds and feces were determined by oxygen bomb calorimetry. The crude ash contents were determined by the high-temperature burning method (550°C, 16 h). The yttrium contents of the feeds and feces samples was determined by inductively coupled plasma emission spectroscopy. The amino acid composition of the raw materials was determined using an automated amino acid analyser after 22 h of hydrolysis with 6 N HCI acid at 110°C (Hitachi L-8800, Tokyo Japan). The protein macromolecules were determined by sodium dodecyl sulphate-polyacrylamide gel electrophoresis (SDS-PAGE) with reference to Bian’s method (40).

### 2.5 Biochemical indexes analyses

The amylase, lipase, and trypsin in the foregut (Nanjing Jiancheng Bioengineering Institute, Nanjing, China), and diamine oxidase activity and endothelin content (Shanghai Enzyme-Linked Biotechnology Co., LTD., Shanghai, China) in serum were measured with commercial kits, according to the manufacturer’s instructions.

### 2.6 Histological observation

The hindguts were placed in a 4% formaldehyde solution, and then dehydrated with different gradients of ethanol, For the exact production process, refer to the method of Zhang et al. (41). Finally, the morphological structure of the sectioned intestine was viewed and photographed using a fluorescence-inverted microscope (Nikon Eclipse Ti-E, Nikon, Japan).

### 2.7 Gene expression analysis

Total hindgut RNA was extracted using the TransZol Up Plus RNA kit (TransGen Biotech, Beijing, China) according to the instructions. Total RNA integrity was verified by 1% agarose gel electrophoresis, and the concentration of the total RNA was measured by a spectrophotometer. RNA was reverse transcribed to cDNA using Prime Script™ RT (Takara, Japan) according to the kit instructions. Quantitative real-time PCR (qRT-PCR) reactions were performed using SYBR^®^ Green Premix *Pro Taq HS qPCR Kit II* (Accurate Biology, China). *Actb* was used as an internal reference gene, and the relative expression levels of the target genes were calculated according to the 2^-ΔΔCT^ calculation method (42). Gene primer sequences are detailed in the Table 3.

**Table 3:** Primer sequence for real-time quantitative PCR.

Gene	Primer sequence (5′ – 3′)		Amplicon size (bp)	Accession no.
*tnf-α*	F	TAGAAGGCAGCGACTCAA	135	NM_001279533.1
	R	CCTGGCTGTAGACGAAGT		
*il-1β*	F	GACAGCCAAAAGAGGAGC	136	XM_019365844.2
	R	TCTCAGCGATGGGTGTAG		
*il-6*	F	ATAGCAAGCATCTACACGCATCTCC	92	XM_003453898.2
	R	GGGCTGCCAGGGAATTGTAAGTC		
*il-8*	F	GCACTGCCGCTGCATTAAG	85	NM_001279704.1
	R	GCAGTGGGAGTTGGGAAGAA		
*il-10*	F	CTGCTAGATCAGTCCGTCGAA	94	XM_013269189.3
	R	GCAGAACCGTGTCCAGGTAA		
*tgf-β1*	F	TGCGGCACCCAATCACACAAC	105	XM_025897821.1
	R	GTTAGCATAGTAACCCGTTGGC		
*cldn*	F	GTCTGTTTCTGGGCGTGGTGTC	84	XM_019367708.2
	R	ACTCCGACTGACTCCTCATCTTCC		
*ocln*	F	GGAGGAAAGCCGCAGTGTTCAG	145	XM_025899615.1
	R	GTCGTAGGCATCGTCATTGTAGGAG		
*zo-1*	F	ACATCGTGCGCTCCAACCAT	123	XM_019358174
	R	GGCTGGACTGTGCTTGTGGT		
*actb*	F	CCACACAGTGCCCATCTACGA	111	XM_003443127.5
	R	CCACGCTCTGTCAGGATCTTCA		

*tgf-α*: tumour necrosis factor-alpha; *il-1β*: interleukin-1 beta; *il-6*: interleukin-6; *il-8*: interleukin-8; *il-10*: interleukin-10; *tgf-β1*: transforming growth factor beta 1; *cldn*: claudin; *ocln*: occludin; *zo-1*: zonula occludens protein-1. *actb*: actin beta.

### 2.8 Intestinal microbiota analysis

Tilapia hindgut samples were sent to Guangzhou Genedenovo Biotechnology Co., Ltd. (Guangzhou, China). Intestinal microbiota total DNA was extracted by using the HiPure Soil DNA Extraction Kit (Magen, Guangzhou China). After quality testing using an ultraviolet (UV)-spectrophotometer (Thermo Fisher Scientific, United States). Subsequently, the V_3_-V_4_ region of the bacterial 16S rRNA gene fragment was amplified using universal primers, and the procedure was referred to Liu et al. (43).

### 2.9 Data calculations

The parameters were calculated according to the following formulae:

Weight gain (WG) = (W1 − W0)/W0.

Feed intake (FI, %) = F/[(W1 + W0)/2]/d × 100.

Special growth rate (SGR, %/d) = (lnW1 − lnW0)/d × 100.

Feed conversion ratio (FCR) = F/(W1 − W0).

Survival rate (SR, %) = N1/N0 × 100.

Protein efficiency ratio (PER) = (W1 − W0)/(F × P).

Apparent digestibility coefficient of nutrients (%) = 100 × [1– (dietary Y_2_O_3_ level/feces Y_2_O_3_ level) × (feces nutrient level/dietary nutrient level)].

Apparent digestibility coefficient of dry matter (%) = 100 × [1– (dietary Y_2_O_3_ level/feces Y_2_O_3_ level)].

Where W1, W0, N1, N0, F, d, and P are the final average weight (g), initial average weight (g), final fish number, initial fish number, feed intake per fish (g), feeding days and crude protein content of feed, respectively.

### 2.10 Statistical analysis

All data after confirming normality and homogeneity. To assess the effect of two factors (Replacement level (RL) and fermentation or not (F)), a two-way ANOVA was first performed (except for the control group). A further one-way ANOVA was then performed for SM or FSM, respectively, with Duncan’s multiple range test. Finally, independent samples t-tests were performed on the equal-alternative groups. Differences were considered significant at the *p < 0.05* level. SPSS 25.0 (Chicago, IL, United States) was used for statistical calculations.

## 3 Results

### 3.1 Growth performance

There was no significant difference in SR when PKC replaced WB (*p* > 0.05, Table 4). FBW, WG, SGR, and PER were significantly lower in the 100% PKC group than in the 0% ~ 60% PKC group (*p* < 0.05). FI and FCR were significantly higher in the 100% PKC group than in the 0% ~ 60% PKC group (*p* < 0.05). As the level of FPKC substituted WB increased, FBW, WG, SGR, and PER showed an increasing and then decreasing trend, reaching a maximum of 40% (*p* < 0.05). FI, FCR, and SGR in the FPKC substituted group were not significantly different from those in the control group (*p* > 0.05). FBW, WG, and SGR were significantly higher in the 60 to 100% FPKC group than in the PKC group. There was no significant interaction between RL and F on the tilapia growth performance (*p* > 0.05).

**Table 4:** Effect of replacing WB with PKC and FPKC on growth performance and feed utilization of tilapia.

Item	Final body weight (g)	Weight gain	Feed intake (%)	Specific growth rate (%/d)	Feed conversion ratio	Protein efficiency ratio	Survival rate (%)
Replacement level of WB with PKC							
0%	62.00 ± 1.21^a^	7.86 ± 0.17^a^	2.71 ± 0.02^b^	3.52 ± 0.03^a^	1.04 ± 0.01^b^	3.21 ± 0.04^a^	98.09 ± 0.95
20%	61.53 ± 2.49^a^	7.83 ± 0.34^a^	2.73 ± 0.05^b^	3.51 ± 0.06^a^	1.05 ± 0.02^b^	3.15 ± 0.08^ab^	98.10 ± 1.90
40%	60.27 ± 0.47^a^	7.62 ± 0.07^a^	2.73 ± 0.02^b^	3.48 ± 0.01^a^	1.05 ± 0.01^b^	3.13 ± 0.03^ab^	100.00 ± 0.00
60%	60.27 ± 0.66^a^	7.61 ± 0.10^a^	2.76 ± 0.01^b^	3.47 ± 0.02^a^	1.06 ± 0.00^b^	3.13 ± 0.01^ab^	99.05 ± 0.95
80%	59.38 ± 0.29^ab^	7.49 ± 0.04^ab^	2.85 ± 0.06^ab^	3.45 ± 0.01^a^	1.10 ± 0.02^b^	3.00 ± 0.07^bc^	97.14 ± 1.65
100%	55.67 ± 1.19^b^	6.96 ± 0.17^b^	2.98 ± 0.08^a^	3.34 ± 0.03^b^	1.17 ± 0.04^a^	2.85 ± 0.09^c^	99.05 ± 0.95
Replacement level of WB with FPKC							
0%	62.00 ± 1.21^y^	7.86 ± 0.17^y^	2.71 ± 0.02^xy^	3.52 ± 0.03^y^	1.04 ± 0.01^xy^	3.21 ± 0.04^xy^	98.09 ± 0.95^xy^
20%	63.97 ± 0.63^y^	8.15 ± 0.09^y^	2.61 ± 0.04^xy^	3.57 ± 0.01^y^	0.99 ± 0.02^xy^	3.33 ± 0.05^xy^	99.05 ± 0.95^x^
40%	72.01 ± 4.20^x^	9.29 ± 0.60^x^	2.51 ± 0.10^y^	3.75 ± 0.09^x^	0.94 ± 0.05^y^	3.55 ± 0.19^x^	93.34 ± 0.95^xy†^
60%	66.16 ± 1.76^xy†^	8.46 ± 0.25^xy†^	2.62 ± 0.03^xy†^	3.62 ± 0.04^xy†^	0.99 ± 0.02^xy†^	3.36 ± 0.05^xy†^	95.24 ± 1.90^xy^
80%	67.02 ± 1.86^xy†^	8.59 ± 0.26^xy†^	2.83 ± 0.14^x^	3.65 ± 0.04^xy†^	1.07 ± 0.05^x^	3.09 ± 0.14^y^	87.62 ± 6.67^y^
100%	67.34 ± 1.01^xy†^	8.64 ± 0.15^xy†^	2.72 ± 0.07^xy^	3.65 ± 0.02^xy†^	1.02 ± 0.03^xy†^	3.23 ± 0.08^xy†^	90.47 ± 3.43^xy^
Two-factors ANOVA							
RL	0.188	0.191	0.012	0.162	0.009	0.016	0.203
F	<0.001	<0.001	0.003	<0.001	<0.001	<0.001	0.004
RL × F	0.093	0.084	0.515	0.071	0.364	0.386	0.311

Data are expressed as mean ± SEM (*n* = 3). ^a,b,c^ Means in the same column with different superscript letters are significantly different (*p* < 0.05) for groups with PKC. ^x,y^ Means in the same column with different superscript letters are significantly different (*p* < 0.05) for groups with FPKC. ^†^*p* < 0.05, FPKC versus PKC for diets with the same replacement level.

### 3.2 Digestive enzyme activity

Lipase and trypsin were significantly lower in the 100% PKC group than in the 0–60% PKC group (*p* < 0.05, Table 5). There was no significant difference in PKC on amylase (*p* > 0.05). As the level of FPKC substituted WB increased, amylase and trypsin tended to increase and then decrease, reaching a significant maximum at 40% (*p* < 0.05). There was no significant difference in FPKC against lipase (*p* > 0.05). amylase, lipase, and trypsin were significantly higher in the 100% FPKC group than in the 100% PKC group (*p* < 0.05). There was a significant interaction effect of RL and F on lipase (*p* > 0.05).

**Table 5:** Effect of replacing WB with PKC and FPKC on digestive enzyme activity of tilapia.

Item	Amylase (U/mgprotein)	Lipase (U/gprotein)	Trypsin (U/mgprotein)
Replacement level of WB with PKC			
0%	8.56 ± 0.96	1.23 ± 0.12^ab^	259.15 ± 9.90^a^
20%	7.53 ± 1.41	1.32 ± 0.01^a^	255.40 ± 29.30^a^
40%	6.91 ± 0.77	1.29 ± 0.07^ab^	263.24 ± 14.82^a^
60%	6.36 ± 0.50	1.12 ± 0.04^ab^	258.64 ± 3.74^a^
80%	7.50 ± 0.75	1.02 ± 0.15^bc^	218.27 ± 2.14^ab^
100%	7.72 ± 0.66	0.75 ± 0.04^c^	207.37 ± 5.59^b^
Replacement level of WB with FPKC			
0%	8.56 ± 0.96^z^	1.23 ± 0.12	259.15 ± 9.90^y^
20%	10.34 ± 0.79^yz^	1.24 ± 0.08	279.20 ± 20.73^xy^
40%	15.05 ± 1.69^x†^	1.18 ± 0.21	305.97 ± 0.43^x†^
60%	10.09 ± 1.49^yz^	1.33 ± 0.05^†^	264.75 ± 2.59^xy^
80%	11.86 ± 0.50^xyz†^	1.28 ± 0.05	276.66 ± 19.98^xy^
100%	12.88 ± 0.85^xy†^	1.28 ± 0.06^†^	243.04 ± 1.85^y†^
Two-factors ANOVA			
RL	0.100	0.082	0.006
F	<0.001	0.014	<0.001
RL × F	0.139	0.018	0.437

Data are expressed as mean ± SEM (*n* = 3). ^a,b,c^ Means in the same column with different superscript letters are significantly different (*p* < 0.05) for groups with PKC. ^x,y,z^ Means in the same column with different superscript letters are significantly different (*p* < 0.05) for groups with FPKC. ^†^*p* < 0.05, FPKC versus PKC for diets with the same replacement level.

### 3.3 Apparent digestibility

The apparent digestibility of dry matter and energy was significantly lower in the PKC than in the 0% PKC group for each substitution group (*p* < 0.05, Table 6), however, the opposite trend was observed in the FPKC group (*p* < 0.05). there was no significant difference in the apparent digestibility of crude protein and crude protein between the PKC and FPKC groups (*p* > 0.05). There was no significant difference between PKC and FPKC groups for crude protein and crude lipid apparent digestibility (*p* > 0.05). 20% ~ 100% FPKC group had significantly higher apparent digestibility of dry matter and energy than the PKC group (*p* < 0.05). there was a significant interaction effect of RL and F on the apparent digestibility of dry matter, energy, and crude lipid (*p* < 0.05).

**Table 6:** Effect of replacing WB with PKC and FPKC on nutrient apparent digestibility of tilapia.

Item	Dry matter (%)	Energy (%)	Crude protein (%)	Crude lipid (%)
Replacement level of WB with PKC				
0%	69.43 ± 0.01^a^	81.45 ± 0.02^a^	85.92 ± 1.00	85.63 ± 3.10
20%	68.63 ± 0.01^b^	81.35 ± 0.02^b^	85.92 ± 0.95	83.34 ± 0.77
40%	65.36 ± 0.03^e^	79.45 ± 0.02^e^	83.16 ± 1.15	83.79 ± 0.45
60%	67.53 ± 0.02^c^	80.18 ± 0.02^c^	84.76 ± 1.25	87.09 ± 0.75
80%	65.93 ± 0.03^d^	79.77 ± 0.01^d^	83.62 ± 1.41	87.21 ± 0.98
100%	62.37 ± 0.01^f^	77.43 ± 0.02^f^	82.49 ± 1.59	84.05 ± 0.71
Replacement level of WB with FPKC				
0%	69.43 ± 0.01^z^	81.45 ± 0.02^z^	85.92 ± 1.00	85.63 ± 3.10
20%	72.19 ± 0.01^w†^	83.19 ± 0.01^w†^	84.88 ± 0.96	84.30 ± 0.26
40%	70.91 ± 0.03^x†^	82.53 ± 0.02^x†^	84.11 ± 1.31	84.42 ± 0.58
60%	73.51 ± 0.02^v†^	83.71 ± 0.02^v†^	85.41 ± 1.18	85.25 ± 0.80
80%	70.42 ± 0.01^y†^	82.16 ± 0.02^y†^	84.13 ± 1.60	85.65 ± 0.46
100%	73.93 ± 0.01^u†^	84.14 ± 0.01^u†^	86.54 ± 0.81	88.40 ± 0.61^†^
Two-factors ANOVA				
RL	<0.001	<0.001	0.579	<0.001
F	<0.001	<0.001	0.209	0.244
RL × F	<0.001	<0.001	0.378	<0.001

Data are expressed as mean ± SEM (*n* = 3). ^a,b,c^ Means in the same column with different superscript letters are significantly different (*p* < 0.05) for groups with PKC. ^x,y,z^ Means in the same column with different superscript letters are significantly different (*p* < 0.05) for groups with FPKC. ^†^*p* < 0.05, FPKC versus PKC for diets with the same replacement level.

### 3.4 Intestinal morphology

**Figure 1:**
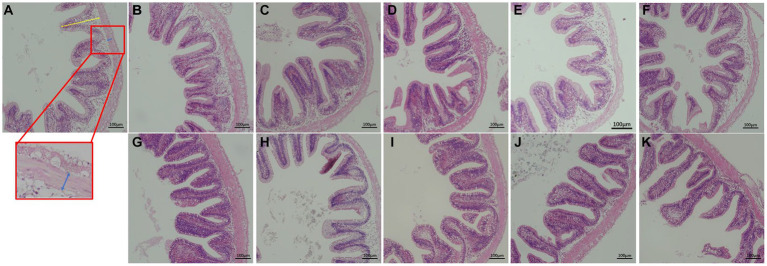
Effect of replacing WB with PKC and FPKC on hindgut histology of tilapia. **(A)** control; **(B)** 20% PKC; **(C)** 40% PKC; **(D)** 60% PKC; **(E)** 80% PKC; **(F)** 100% PKC; **(G)** 20% FPKC; **(H)** 40% FPKC; **(I)** 60% FPKC; **(J)** 80% FPKC; **(K)** 100% FPKC; Blue arrows: muscular thickness (μm); Yellow arrows: villus height (μm); The magnification was ×200, and the minimum scale (lower right) was 100 μm.

Muscularis thickness was significantly lower in the 100% PKC group than in the 0 and 20% PKC groups (*p* < 0.05, Table 7 and Figure 1). villus height was significantly lower in the 100% PKC group than in the 0% ~ 80% PKC group (*p* < 0.05). villus height was significantly higher in the FPKC substitution groups than in the 0% FPKC group (*p* < 0.05). There was no significant difference in the effect of FPKC on muscular thickness (*p* > 0.05). villus height was significantly higher in the 20, 80, and 100% FPKC groups than in the PKC group (*p* < 0.05). There was no significant interaction effect of RL and F on muscular thickness and villus height (*p* > 0.05).

**Table 7:** Effect of replacing WB with PKC and FPKC on the intestinal histomorphology of tilapia.

Item	Muscularis thickness (μm)	Villus height (μm)
Replacement level of WB with PKC		
0%	49.50 ± 0.50^a^	246.87 ± 4.07^a^
20%	48.17 ± 2.03^a^	253.46 ± 13.07^a^
40%	46.75 ± 1.45^ab^	256.90 ± 0.18^a^
60%	46.40 ± 1.58^ab^	260.58 ± 16.65^a^
80%	43.26 ± 3.14^ab^	257.09 ± 5.23^a^
100%	39.41 ± 3.62^b^	208.04 ± 3.40^b^
Replacement level of WB with FPKC		
0%	49.50 ± 0.50	246.87 ± 4.07^z^
20%	56.29 ± 5.39	325.90 ± 12.66^x†^
40%	51.64 ± 5.00	313.73 ± 14.71^xy^
60%	51.87 ± 2.97	306.81 ± 9.60^xy^
80%	50.30 ± 0.73	310.62 ± 3.42^xy†^
100%	50.63 ± 2.38	284.05 ± 3.99^y†^
Two-factors ANOVA		
RL	0.245	0.002
F	0.002	<0.001
RL × F	0.866	0.526

Data are expressed as mean ± SEM (*n* = 3). ^a,b,c^ Means in the same column with different superscript letters are significantly different (*p* < 0.05) for groups with PKC. ^x,y,z^ Means in the same column with different superscript letters are significantly different (*p* < 0.05) for groups with FPKC. ^†^*p* < 0.05, FPKC versus PKC for diets with the same replacement level.

### 3.5 Intestinal mucosal barrier status

**Figure 2:**
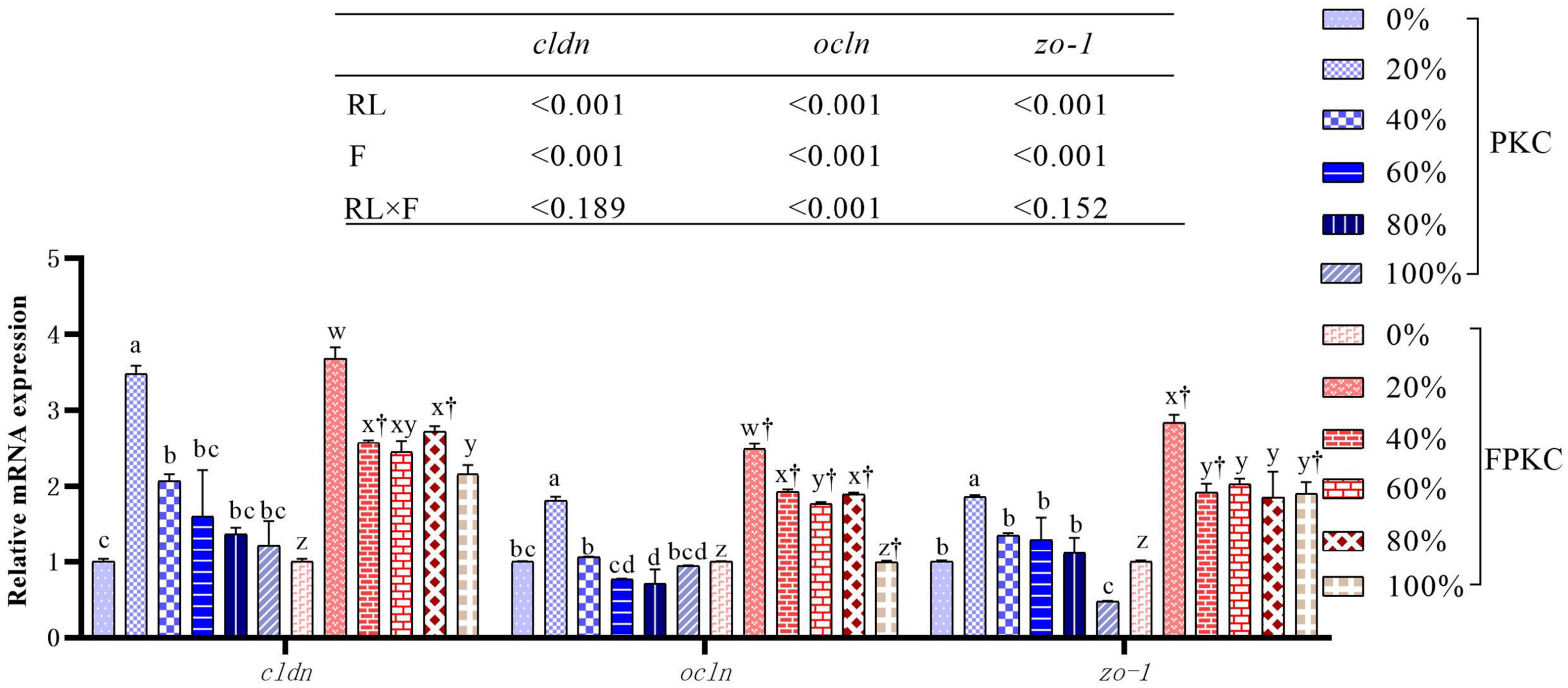
Effect of replacing WB with PKC and FPKC on intestinal tight junction-related gene expression of tilapia. ^a,b,c^ Means in the same bars with different superscript letters are significantly different (*p* < 0.05) for groups with PKC; ^x,y,z^ Means in the same bars with different superscript letters are significantly different (*p* < 0.05) for groups with FPKC; ^†^*p* < 0.05, FPKC versus PKC for diets with the same replacement level; *cldn: claudin*; *ocln*: *occludin; zo-1*: zona occludns protein 1.

Diamine oxidase and endothelin were significantly lower in the 20% PKC group than in the 0, 80, and 100% PKC groups (*p* < 0.05, Table 8). The FPKC substitution groups had significantly lower diamine oxidase than the 0%FPKC group (*p* < 0.05). Endothelin was significantly lower in the 20% FPKC group than in the 0% FPKC group and each of the substitution groups (*p* < 0.05). There was a significant interaction effect of RL and F on diamine oxidase (*p* < 0.05). With the increase of PKC and FPKC replacement WB levels, *cldn*, *ocln*, and *zo-1* mRNA expression levels showed a trend of increasing and then decreasing, and all of them reached the maximum at 20% (*p* < 0.05, Figure 2). *cldn*, *ocln*, and *zo-1* mRNA expression levels were significantly higher in the 40% FPKC group than in the 40% PKC group (*p* < 0.05). There was a significant interaction effect of RL and F on ocludin (*p* < 0.05).

**Table 8:** Effect of replacing WB with PKC and FPKC on intestinal permeability of tilapia.

Item	Diamine oxidase (U/mL)	Endothelin (ng/mL)
Replacement level of WB with PKC		
0%	22.49 ± 1.11^a^	54.01 ± 0.40^a^
20%	13.06 ± 0.57^b^	52.35 ± 0.29^b^
40%	13.81 ± 0.66^b^	53.68 ± 0.67^ab^
60%	21.11 ± 0.90^a^	53.68 ± 0.29^ab^
80%	22.85 ± 0.88^a^	54.57 ± 0.57^a^
100%	20.16 ± 1.94^a^	55.01 ± 0.22^a^
Replacement level of WB with FPKC		
0%	22.49 ± 1.11^w^	54.01 ± 0.40^x^
20%	12.02 ± 0.21^z^	51.13 ± 0.59^y^
40%	15.52 ± 0.16^y^	52.90 ± 0.38^x^
60%	18.18 ± 1.00^xy^	53.24 ± 0.51^x^
80%	16.56 ± 0.74^xy†^	53.12 ± 0.22^x^
100%	19.56 ± 1.54^x^	53.23 ± 0.57^x†^
Two-factors ANOVA		
RL	<0.001	<0.001
F	0.009	<0.001
RL × F	0.010	0.628

Data are expressed as mean ± SEM (*n* = 3). ^a,b,c^ Means in the same column with different superscript letters are significantly different (*p* < 0.05) for groups with PKC. ^x,y,z^ Means in the same column with different superscript letters are significantly different (*p* < 0.05) for groups with FPKC. ^†^*p* < 0.05, FPKC versus PKC for diets with the same replacement level.

### 3.6 Intestinal immune status

**Figure 3:**
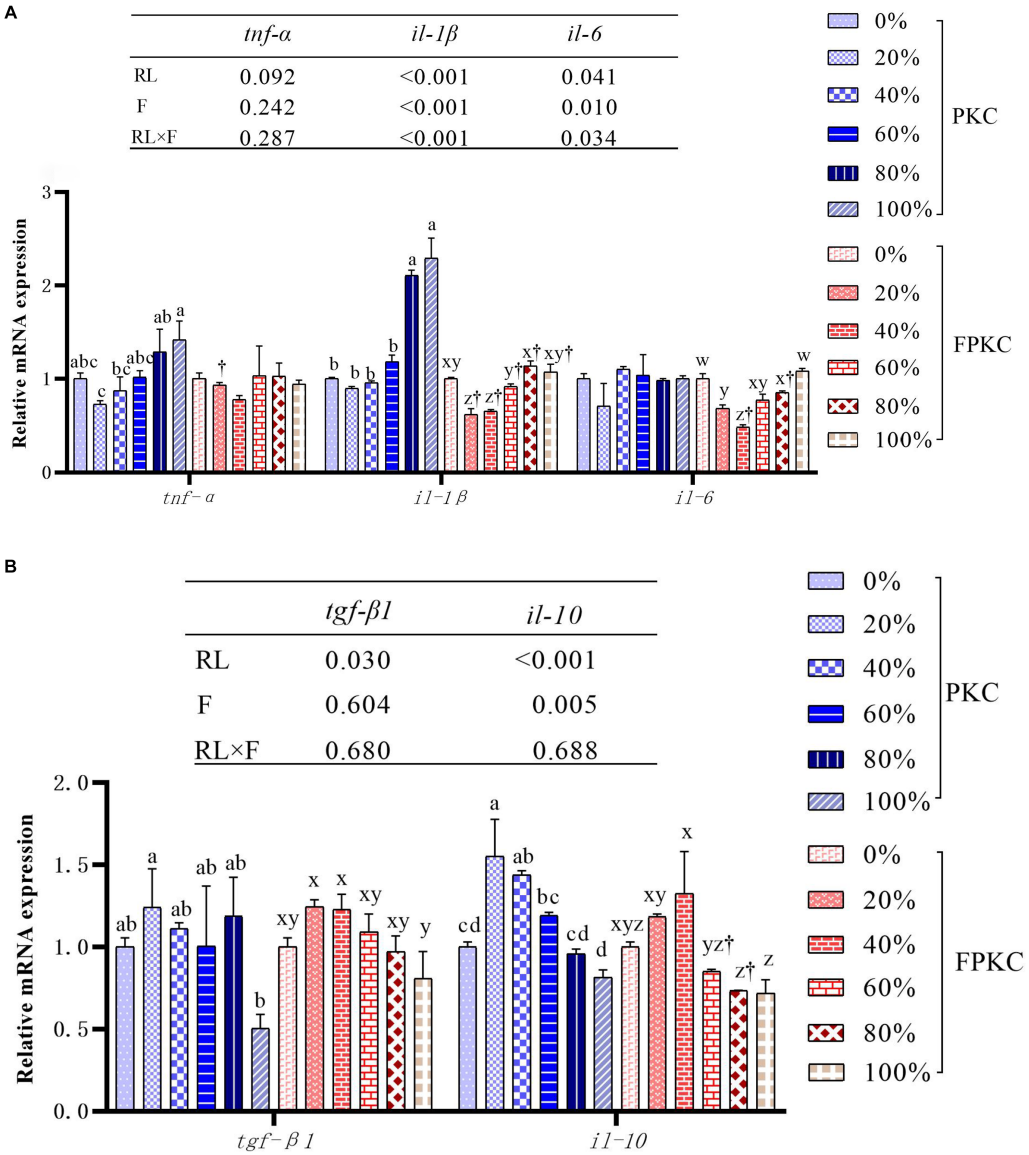
Effect of replacing WB with PKC and FPKC on intestinal inflammation-related gene expression of tilapia. Data are expressed as mean ± SEM (*n* = 3); ^a,b,c^ Means in the same bars with different superscript letters are significantly different (*p* < 0.05) for groups with PKC; ^x,y,z^ Means in the same bars with different superscript letters are significantly different (*p* < 0.05) for groups with FPKC; ^†^*p* < 0.05, FPKC versus PKC for diets with the same replacement level; *tnf-α*: tumour necrosis factor alpha; *il-1β*: interleukin-1 beta; *il-6*: interleukin- 6; *il-8*: interleukin-8; *tgf-β1*: transforming growth factor β1; *il-10*: interleukin-10.

The *tnf-α*, il-6, *tgf-β1* and *il-10* mRNA expression in the PKC replacement group was not significantly different from that of 0% PKC (*p* > 0.05, Figures 3A,B). 80% ~ 100% PKC significantly up-regulated *il-1β* mRNA expression level (*p* < 0.05). FPKC substitution level lower than 40% down-regulated *il-1β* and *il-6* mRNA expression levels (*p* < 0.05), and *tnf-α*, *tgf-β1* and *il-10* mRNA expression was not significantly different from the 0% FPKC group (*p > 0.05*). *il-1β* mRNA expression levels were significantly lower in each FPKC substitution group than in the PKC group (*p* < 0.05). RL and F had significant interaction effects on *il-1β* and *il-6* mRNA expression (*p* < 0.05).

### 3.7 Intestinal microbiota

#### 3.7.1 α-diversity analysis

Chao1 and Ace indices were significantly lower in the 40% PKC group than in the 0% PKC group (*p* < 0.05, Table 9). There were no significant differences in Shannon and Simpson for PKC (*p* > 0.05). Chao1 and Ace were significantly lower in the 40% and 100% FPKC than in the control group (*p* < 0.05). Simpson was significantly higher in the 100% FPKC was significantly higher than that of the 0% FPKC group (*p* < 0.05). Shannon and Simpson were significantly higher in 100% FPKC than that in 100% PKC (*p* < 0.05). RL and F had no significant interaction effect on Shannon, Simpson, Chao1, and Ace (*p* > 0.05).

**Table 9:** Effect of replacing WB with PKC and FPKC on alpha-diversity index of tilapia intestinal flora.

	Shannon	Simpson	Chao1	Ace	Goods coverage
Replacement level of WB with PKC					
0%	2.69 ± 0.26	0.67 ± 0.05	531.21 ± 50.65^a^	547.43 ± 47.37^a^	1.00
40%	2.82 ± 0.27	0.68 ± 0.05	395.72 ± 28.49^b^	412.94 ± 19.76^b^	1.00
100%	2.40 ± 0.12	0.65 ± 0.03	421.83 ± 21.75^ab^	429.90 ± 31.57^ab^	1.00
Replacement level of WB with FPKC					
0%	2.69 ± 0.26	0.67 ± 0.05^y^	531.21 ± 50.65^x^	547.43 ± 47.37^x^	1.00
40%	2.93 ± 0.20	0.68 ± 0.02^xy^	383.27 ± 10.71^y^	398.27 ± 10.65^y^	1.00
100%	3.40 ± 0.23^†^	0.78 ± 0.01^x†^	376.73 ± 40.57^y^	402.19 ± 43.98^y^	1.00
Two-factors ANOVA					
RL	0.899	0.345	0.732	0.731	0.721
F	0.031	0.077	0.328	0.490	0.067
RL × F	0.067	0.067	0.570	0.829	0.079

Data are expressed as mean ± SEM (*n* = 3). ^a,b,c^ Means in the same column with different superscript letters are significantly different (*p* < 0.05) for groups with PKC. ^x,y^ Means in the same column with different superscript letters are significantly different (*p* < 0.05) for groups with FPKC. ^†^*p* < 0.05, FPKC versus PKC for diets with the same replacement level.

#### 3.7.2 Microbial composition

**Figure 4:**
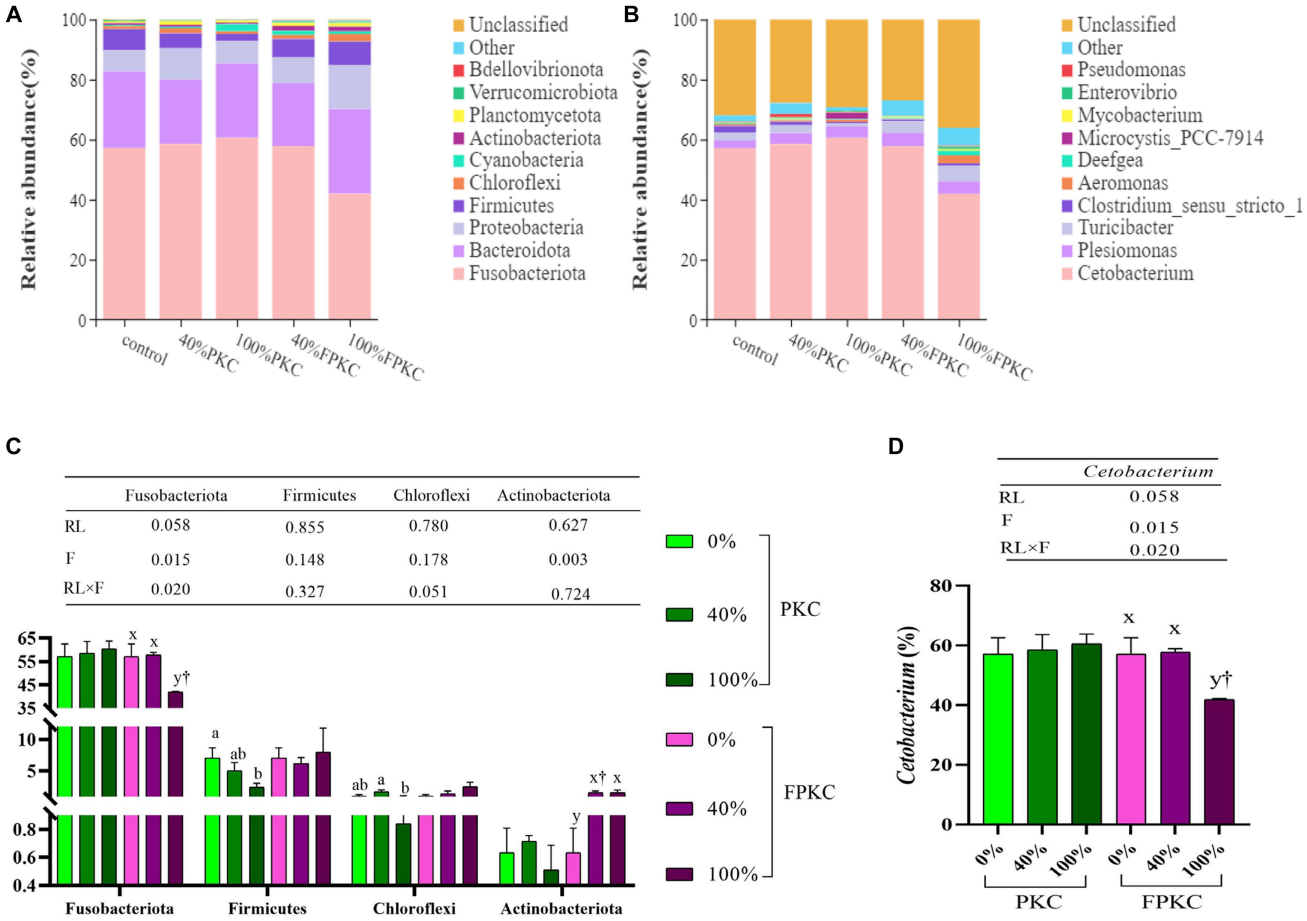
Effect of replacing with PKC and FPKC on intestinal microbial of tilapia. **(A)** phylum level; **(B)** genus level; **(C)** Relative abundance is significantly different at the phylum level (Top 10); **(D)** Relative abundance is significantly different at the genus level (Top 10). Data are expressed as mean ± SEM (*n* = 3); ^a,b,c^ Means in the same bars with different superscript letters are significantly different (*p* < 0.05) for groups with PKC; ^x,y^ Means in the same bars with different superscript letters are significantly different (*p* < 0.05) for groups with FPKC; ^†^*p* < 0.05, FPKC versus PKC for diets with the same replacement level.

The dominant groups of tilapia gut microorganisms at the phylum level were Fusobacteriota, Bacteroidota, Proteobacteria, and Firmicutes (Figure 4A). The top 10 dominant genera in terms of relative abundance at the phylum level were analyzed for differences, and there were no significant differences between Fusobacteriota and Actinobacteriota in the PKC group (*p* > 0.05, Figure 4C). Firmicutes were significantly lower in 100% PKC than in 0% PKC (*p* < 0.05). Chloroflexi was significantly lower in the 100% PKC than in the 20% PKC group (*p* < 0.05). Fusobacteriota was significantly lower in 100% FPKC than in the 0, 20% FPKC, and 100% PKC groups (*p* < 0.05). Actinobacteriota was significantly higher in 20 and 100% FPKC than in 0% FPKC (*p* < 0.05). RL and F had a significant interaction effect on Fusobacteriota (*p* < 0.05).

The top 10 dominant genera in terms of relative abundance at the genus level were analyzed for differences (Figure 4B). *Cetobacterium* was significantly lower in the 100% FPKC than in the 0 and 40% FPKC groups (*p* < 0.05, Figure 4D). There was no significant difference in *Cetobacterium* by PKC (*p* > 0.05). *Cetobacterium* was significantly lower in the 100% FPKC group than in the 100% PKC group (*p* < 0.05). RL and F had a significant interaction effect on *Cetobacterium* (*p* < 0.05).

## 4 Discussion

In this study, complete replacement of WB with PKC negatively affected the growth of tilapia. Ng and Chen (7) reported that dietary supplementation with 20% PKC had no significant effect on the growth performance of Hybrid Asian-African Catfish. In contrast, supplementation with more than 20% palm meal decreased the growth and utilisation efficiency. Studies involving Nile tilapia have shown that dietary addition of 25% PKC did not affect its growth; however, the growth and food conversion rate declined when the PKC concentration was increased (6). A high degree of PKC substitution reduced the growth performance of tilapia due to the high levels of indigestible NSPS (19, 20). NSPS are sticky and adhere to the digestive tract, thus hindering nutrient absorption (5), Numerous studies have shown that the addition of high levels of NSPs to feed reduced the utilisation of nutrients and decreased the growth of fish (44, 45). In contrast, the replacement of WB with FPKC improved the growth performance of tilapia. Optimal growth was observed at a substitution level of 40%. Further, weight gain and specific growth rate were significantly higher in the FPKC group than in the PKC group at 60 to 100% substitution levels. This result was consistent with the findings of Wattanakul et al. (8), who reported that the addition of 12.5% FPKC to red tilapia (*Oreochromis niloticus × O. mossambicus*) feed yielded the best growth performance and feed utilisation. These results suggest that FPKC is a better alternative to WB than PKC. Studies have shown that fermentation not only degrades macromolecules completely (Supplementary Figure S1) but also generates useful metabolites, degrades anti-nutritional factors, improves feed flavour and palatability, promotes animal feed intake, and increases feed nutritional value and feed utilisation (31), Thus, the growth performance of tilapia can be improved. However, Ng et al. (18) reported that the growth of red hybrid tilapia was reduced by the addition of 20% and 40% fermented palm meal to red hybrid tilapia feed. However, this finding was inconsistent with the results of the present study and may be related to the fermentation of the raw material (46).

The activity of intestinal digestive enzymes determines the efficiency of feed nutrient utilisation and growth rate of fish (47, 48). In the study, the complete replacement of WB with PKC significantly reduced the activities of lipase and trypsin, which were highly consistent with the growth performance, suggesting that high degree of replacement with PKC may inhibit the growth of tilapia by decreasing intestinal digestion and absorption. This may be related to the high levels of NSPS (19, 20). The impact of NSPs on the activity of intestinal digestive enzymes is strongly related to their physico-chemical properties. Some soluble non-starch polysaccharides (SNSPs) can increase the viscosity of chyme, thereby inhibiting the activity of digestive enzymes (49), NSPs may also reduce the activity of digestive enzymes by binding or by wrapping around the substrate (50). Amylase activity was unaffected by the concentration of PKC-substituted WB, suggesting that this omnivorous fish has an adequate number of metabolic pathways to digest carbohydrates (8). However, FPKC increased the activity of tilapia digestive enzymes, especially amylase, which was consistent with the findings of Wattanakul et al. (8) who found that FPKC increased the digestive enzyme activity of red tilapia. Fermentation degrades the anti-nutritional factors in the feed, and the macromolecules are transformed into small molecules that can be easily absorbed by the animals (Supplementary Figure S1) (51).

The apparent digestibility of nutrients reflects the degree of digestion in the fish, and thus indirectly indicates the activity of digestive enzymes (52). The apparent digestibility of dry matter and the energy were significantly reduced by the addition of PKC to red hybrid tilapia feeds (18). This finding is consistent with the results of the present study, where the apparent digestibility of dry matter and the energy decreased linearly with increasing levels of PKC substitution. This is consistent with the changes in the activity of the intestinal digestive enzymes. The reduction in apparent digestibility of dry matter and the energy can be attributed to undigested cellulose in plant proteins (53). PKC contains about 12% cellulose (20). However, plant-based ingredients, due to their high cellulose content, may accelerate the movement of chyme through the gut, thus reducing the apparent digestibility of dry matter and the energy in fish feed (54). Conversely, the FPKC substitution of WB significantly increased the apparent digestibility of dry matter and the energy compared with that of the PKC. This is inconsistent with the findings of Ng et al. (18) who found that in red hybrid tilapia feed, the apparent digestibility of dry matter and energy was significantly reduced with the addition of fermented or enzyme-treated PKC. This may be related to the processing of the raw material (46).

Feed ingredients inevitably affect the development and morphology of the fish gut, and therefore gut morphology is often used to assess the potential physiological effects of feeds on fish (55). In this study, the complete replacement of WB with PKC significantly altered the gut morphology of tilapia. A decrease in villus height indicates a decrease in the internal digestive area of the intestine, whereas a decrease in muscularis thickness indicates a decrease in intestinal peristalsis. Both of these morphological parameters interfere with intestinal digestion and absorption (56, 57). A high level of PKC substitution inhibited intestinal digestion and absorption, which led to decreased growth performance. However, the replacement of WB by FPKC increased the villus height significantly than in the control group. The degradation of anti-nutritional factors by fermentation alleviated the damage to the intestinal structure induced by PKC in tilapia, thus improving the intestinal health of the fish.

It is well known that gut health is critical to the growth and health of fish. Nonetheless, studies have shown that gut health is influenced by the composition of the diet consumed (58). The intestinal mucosa contains chemical, mechanical, biological, and immune barriers (59). The intestinal mucosal barrier effectively prevents invasion by toxins, antigens, and pathogens (60). It contributes to the maintenance of intestinal health. In general, serum diamine oxidase activity and endothelin concentrations are strong indicators of intestinal mucosal permeability. Elevated concentrations or activities of these indicators suggest intestinal mucosal damage (58, 61). In this study, the substitution of WB with 20% PKC significantly reduced serum diamine oxidase activity and endothelin concentrations. Notably, The fermentation of PKC significantly decreased the diamine oxidase activity compared with the control group. The result indicated that FPKC strongly increased the integrity of intestinal mucosa. In addition, the structural integrity of the intestinal tight junction is closely related to the intestinal mucosal barrier function (62). The levels of *occludin*, *zo-1*, and *claudin* genes, which are related to the tight junction structure, represent the integrity of the intestinal mucosal barrier (63, 64). In this study, the replacement of WB with low levels of PKC significantly up-regulated the expression of *cldn*, *ocln*, and *zo-1*, while high levels of PKC yielded the opposite result. However, FPKC significantly increased the expression of structural genes related to tight junctions (*cldn*, *ocln*, and *zo-1*) in the intestine, suggesting that FPKC is more effective in maintaining the integrity of the intestinal barrier.

The intestinal inflammatory response is strongly related to intestinal health and is generally determined by the expression of pro- and anti-inflammatory factors (42). In this study, PKC replacement had no significant effect on *tnf-α* and *il-6* gene expression. However, *il-1β* is one of the key factors expressed in response to intestinal inflammation. *il-1β*, which is mainly synthesized and secreted by macrophages, is a major pro-inflammatory factor released in early inflammatory response. It is involved in activating the proliferation of T and B cells and promoting the production of inflammatory factors (65). In this study, excessive PKC significantly promoted the expression of the pro-inflammatory factor (*il-1β*) and inhibited the expression of the anti-inflammatory factor (*tgf-β1*), These results may be attributed to the continued intestinal accumulation of anti-nutritional factors present in PKC, which compressed and destroyed the integrity of the intestinal epithelial mucosa, and induced enteritis (66, 67). However, we found that substitution with low levels of FPKC significantly down-regulated the pro-inflammatory factors (*il-6* and *il-1β*). Similarly, the expression of pro-inflammatory factors was inhibited when a small amount of *Micropterus salmoides* feed was replaced with Bacillus aerobic fermented soybean meal (68). We speculate that the fermentation of palm meal may result in the synthesis of oligosaccharides, which can effectively improve the immunity of animals. However, increasing feed replacement can lead to the eventual accumulation of residual anti-nutrient factors and induce adverse reactions.

Intestinal flora maintain gut health and are regulated by diet (69). Alpha diversity indices such as Chao1 and Ace are used to determine the species richness, while Shannon and Simpson indices are used to measure the diversity of microbial flora (70). In this study, the substitution of WB with PKC and FPKC reduced the abundance of intestinal flora in tilapia. Interestingly, however, PKC improved Shannon and Simpson indices after fermentation. The Shannon and Simpson indices of 100% FPKC were significantly higher than those of 100% PKC. Some studies have reported that dietary supplementation of small peptides improved the diversity of intestinal flora in largemouth bass (70). Therefore, FPKC can improve the diversity of intestinal flora, probably due to the presence of a large number of small molecular peptides generated by fermentation.

At the phylum level, the dominant groups in all groups were Fusobacteriota, Bacteroidota, Proteobacteria, and Firmicutes, similar to the results of previous studies (71, 72). Fusobacteria are conditionally pathogenic, Gram-negative bacteria found in water bodies. Their reduced abundance improves lipid utilisation in the host, which in turn improves digestion and absorption and decreases the risk of disease (73). Fusobacteria have also been shown to promote colorectal adenomas (74). In this study, the abundance of Fusobacteriota was significantly lower in the group treated with 100% FPKC than in the control, 20% FPKC, and 100% PKC groups. It indicated that FPKC improved digestion and absorption and reduced the prevalence of tilapia compared with PKC-substituted WB. Firmicutes, as a probiotic, synthesize short-chain fatty acids to supply nutrients for intestinal mucosal cells, which regulate the intestinal microecology (75). The relative abundance of Firmicutes is positively correlated with dietary calorie intake (76). It improves the digestibility of food and immunity of fish and protects them from the destructive effects of bacterial pathogens in the intestine (77, 78). In this study, the abundance of Firmicutes was significantly lower in the 100% PKC group than in the control group. FPKC increased the abundance of Firmicutes, indicating that FPKC affected the nutrient metabolism of gut bacteria in tilapia. Thus, tilapia ingesting FPKC may digest food more easily and exhibit stronger immunity. Chloroflexi exhibits a very high metabolic diversity and also utilises saccharides as substrates for fermentation and degradation of complex organic compounds (79). In this study, no significant difference existed between Chloroflexi in the PKC and FPKC replacement groups and the control group. Actinobateriota produce a wide range of beneficial metabolites, antibiotics, and bioactivities (80). They play a key role in regulating intestinal permeability, immunomodulation, and metabolism (81). Its substantial increase increases the abundance of beneficial substances and facilitates intestinal homeostasis and immune regulation. In this study, FPKC significantly increased the abundance of Actinobacteriota. It suggests that FPKC contributes significantly to the maintenance of intestinal homeostasis and intestinal health. *Cetobacterium*, a member of the Fusobacteria, is a common probiotic found in the intestinal tract of fish. It ferments peptide carbohydrates, which are metabolised to vitamin B_12_, acetic acid, and propionic acid, and thus participate in the regulation of host intestinal microecology and nutrient metabolism (82, 83). In the study, the concentrations of *Cetobacterium* were significantly lower in the 100% FPKC group than in the control, 40% FPKC, and 100% PKC groups. It has been suggested that a decrease in *Cetobacterium* abundance increases the body’s immunity (73). However, a comprehensive study is needed to analyse the specific regulatory mechanisms underlying the effects on the host.

In summary, our results suggest that FPKC decreases the number of pathogenic bacteria and increases the number of beneficial bacteria in the gut. The decrease in the number of pathogenic bacteria in fish may be attributed to the inhibitory effects of volatile fatty acids (VFAs) produced during the fermentation of feed in certain intestinal bacteria (84), increasing the abundance of beneficial bacteria. In contrast, the high levels of NSPs in PKC, which dissolve in water, increase the viscosity of chyme. The decreased flow of chyme in the intestinal tract and the scarcity of oxygen decrease the number of beneficial bacteria (9).

## 5 Conclusion

WB can be replaced with PKC up to 80% in tilapia feeds. However, the high percentage of gluten induced intestinal inflammation, impaired gut health, and reduced dietary nutrient utilisation and growth performance. Complete replacement of WB with FPKC promoted intestinal immunity. It also improved dietary nutrient utilisation and growth performance. However, the optimal growth was achieved at a 40% replacement level.

## Data availability statement

The original contributions presented in the study are publicly available. This data can be found at: https://www.ncbi.nlm.nih.gov/, BioProject: PRJNA1065588.

## Ethics statement

The animal studies were approved by Animal Research and Ethics Committee of Guangdong Ocean University (GDOU-IACUC-2021-A2113). The studies were conducted in accordance with the local legislation and institutional requirements. Written informed consent was obtained from the owners for the participation of their animals in this study.

## Author contributions

HH: Conceptualization, Data curation, Formal analysis, Investigation, Methodology, Software, Supervision, Validation, Writing – original draft, Writing – review & editing. XL: Data curation, Formal analysis, Methodology, Supervision, Writing – review & editing. XM: Data curation, Formal analysis, Methodology, Supervision, Validation, Writing – review & editing. YL: Data curation, Formal analysis, Methodology, Supervision, Validation, Writing – review & editing. JF: Data curation, Formal analysis, Methodology, Supervision, Writing – review & editing. LulZ: Data curation, Formal analysis, Methodology, Supervision, Validation, Writing – review & editing. JC: Data curation, Formal analysis, Methodology, Supervision, Validation, Writing – review & editing. LuZ: Data curation, Formal analysis, Methodology, Supervision, Validation, Writing – review & editing. HM: Data curation, Formal analysis, Methodology, Supervision, Validation, Writing – review & editing. JD: Data curation, Formal analysis, Funding acquisition, Methodology, Project administration, Resources, Supervision, Validation, Visualization, Writing – review & editing.

## References

[1] Huang Q. Consideration of the feed grain supply strategy under China’s food security strategy. China Feed. (2023) 22:15–25. doi: 10.15906/j.cnki.cn11-2975/s.20232202

[2] Yang S Cui X. Large-scale production: a possible way to the balance between feed grain security and meat security in China. J Agric Food Res. (2023) 14:100745. doi: 10.1016/j.jafr.2023.100745

[3] Yang T Liao M Liu S He Y Wu J. Study on feed by mixed fungi fermentation of cassava dregs to replace wheat bran. Feed Rev. (2014) 11:1–5. doi: 10.3969/j.issn.1001-0084.2014.11.001

[4] Wang B Wang Q Guo Z Guo Z Lei M Li G. Effects of wheat bran replacement by tea leaf residue on growth performance, slaughter performance and meat quality of meat rabbits. Chin J Anim Nutr. (2022) 34:1186–93. doi: 10.3969/j.issn.1006⁃267x.2022.02.050

[5] Sharmila A Alimon AR Azhar K Noor HM, Samsudin. A.A. The nutritive value of palm kernel cake for animal feed Mal J Anim Sci (2014) 17:1–18. Available at: http://www.etawau.com/OilPalm/Elaeis_guineensis.htm

[6] Omoregie E Ogbemudia FI. Effect of substituting fish meal with palm kernel meal on growth and food utilization of the Nile tilapia, *Oreochromis niloticus*. Isr J Aquac Bamid. (1993) 45:113–9.

[7] Ng WK Chen ML. Replacement of soybean meal with palm kernel meal in practical diets for hybrid Asian-African catfish, *clarias macrocephalus* × *C. gariepinus*. J Appl Aquac. (2002) 12:67–76. doi: 10.1300/J028v12n04_06

[8] Wattanakul W Thongprajukaew K Hahor W Suanyuk N. Optimal replacement of soybean meal with fermented palm kernel meal as protein source in a fish meal-soybean meal-based diet of sex reversed red tilapia (*Oreochromis niloticus* × *O. mossambicus*). Animals. (2021) 11:2287. doi: 10.3390/ani11082287, PMID: 34438745 PMC8388480

[9] Guo B. Effect of different pretreatment palm kernel cake on pellet characteristic, nutrient digestibility and intestinal microflora of pigs. [Huazhong, China]. Huazhong Agricultural University (2023) 51–53.

[10] Mahlia TMI Ismail N Hossain N Silitonga AS Shamsuddin AH. Palm oil and its wastes as bioenergy sources: a comprehensive review. Environ Sci Pollut Res. (2019) 26:14849–66. doi: 10.1007/s11356-019-04563-x, PMID: 30937750

[11] Alimon AR. The nutritive value of palm kernel cake for animal feed. Palm Oil Dev. (2004) 40:12–4. Available at: https://www.researchgate.net/publication/242540604

[12] da Silva LO de Carvalho GGP Tosto MSL Lima VGO Cirne LGA Pina D. Digestibility, nitrogen metabolism, ingestive behavior and performance of feedlot goats fed high-concentrate diets with palm kernel cake. Livest Sci. (2020) 241:104226. doi: 10.1016/j.livsci.2020.104226

[13] Agunbiade JA Wiseman J Cole DJA. Energy and nutrient use of palm kernels, palm kernel meal and palm kernel oil in diets for growing pigs. Anim Feed Sci Technol. (1999) 80:165–81. doi: 10.1016/S0377-8401(99)00070-X

[14] Rhule SWA. Growth rate and carcass characteristics of pigs fed on diets containing palm kernel cake. Anim Feed Sci Technol. (1996) 61:167–72. doi: 10.1016/0377-8401(95)00934-5

[15] Azizi MN Loh TC Foo HL Teik Chung EL. Is palm kernel cake a suitable alternative feed ingredient for poultry? Animals. (2021) 11:338. doi: 10.3390/ani11020338, PMID: 33572711 PMC7911022

[16] Abdeltawab AM Khattab MSA. Utilization of palm kernel cake as a ruminant feed for animal: a review. N A J Biol Sci. (2018) 11:157–64. doi: 10.3923/ajbs.2018.157.164

[17] Thongprajukaew K Rodjaroen S Yoonram K Sornthong P Hutcha N Tantikitti C. Effects of dietary modified palm kernel meal on growth, feed utilization, radical scavenging activity, carcass composition and muscle quality in sex reversed Nile tilapia (*Oreochromis niloticus*). Aquaculture. (2015) 439:45–52. doi: 10.1016/j.aquaculture.2015.01.021

[18] Ng WK Lim HA Lim SL Ibrahim CO Ng WK. Nutritive value of palm kernel meal pretreated with enzyme or fermented with Trichoderma koningii (*Oudemans*) as a dietary ingredient for red hybrid tilapia (*Oreochromis sp*.). Aquac Res. (2002) 33:1199–207. doi: 10.1046/j.1365-2109.2002.00757.x

[19] Knudsen KEB. Carbohydrate and lignin contents of plant materials used in animal feeding. Anim Feed Sci Technol. (1997) 67:319–38. doi: 10.1016/S0377-8401(97)00009-6

[20] Düsterhöft E Voragen AGJ Engels FM. Non-starch polysaccharides from sunflower (*Helianthus annuus*) meal and palm kernel (*Elaeis guineenis*) meal—preparation of cell wall material and extraction of polysaccharide fractions. J Sci Food Agric. (1991) 55:411–22. doi: 10.1002/jsfa.2740550309

[21] Choct M Annison G. Anti-nutritive effect of wheat pentosans in broiler chickens: roles of viscosity and gut microflora. Br Poult Sci. (1992) 33:821–34. doi: 10.1080/00071669208417524, PMID: 1393677

[22] Sulabo RC Ju WS Stein HH. Amino acid digestibility and concentration of digestible and metabolizable energy in copra meal, palm kernel expellers, and palm kernel meal fed to growing pigs. J Anim Sci. (2013) 91:1391–9. doi: 10.2527/jas2012-5281, PMID: 23307844

[23] Ezieshi EV Olomu JM. Nutritional evaluation of palm kernel meal types: 1. Proximate composition and metabolizable energy values. Afr J Biotechnol. (2007) 6:2484–6. doi: 10.5897/AJB2007.000-2393

[24] Cowey CB. Amino acid requirements of fish: a critical appraisal of present values. Aquaculture. (1994) 124:1–11. doi: 10.1016/0044-8486(94)90349-2

[25] Forster I Ogata HY. Lysine requirement of juvenile Japanese flounder *Paralichthys olivaceus* and juvenile red sea bream *Pagrus major*. Aquaculture. (1998) 161:131–42. doi: 10.1016/S0044-8486(97)00263-9

[26] Ruchimat T Masumoto T Hosokawa H Itoh Y Shimeno S. Quantitative lysine requirement of yellowtail (*Seriola quinqueradiata*). Aquaculture. (1997) 158:331–9. doi: 10.1016/S0044-8486(97)00215-9

[27] Espe M Hevrøy EM Liaset B Lemme A El-Mowafi A. Methionine intake affect hepatic Sulphur metabolism in Atlantic salmon, *Salmo salar*. Aquaculture. (2008) 274:132–41. doi: 10.1016/j.aquaculture.2007.10.051

[28] Olukomaiya O Fernando C Mereddy R Li X Sultanbawa Y. Solid-state fermented plant protein sources in the diets of broiler chickens: a review. Animal Nutr. (2019) 5:319–30. doi: 10.1016/j.aninu.2019.05.005, PMID: 31890908 PMC6920459

[29] Sun Z Mei L Huang X Li Y. Research progress on fermented feed by microbial cooperating with enzyme and its application to animal production. Chin J Anim Sci. (2021) 57:42–7. doi: 10.19556/j.0258-7033.20200828-01

[30] Zhou S Zhang Q Qi X Qi L Sun L Yu D. Study on the method of the synergy bacillus subtilis and neutral protease in soybean meal fermentation. N A Ind. (2016) 37:163–8.

[31] Song G Sun C Yuan X Bao L. Application progress of bacteria-enzyme co-fermentation feed in animal husbandry. Mod J Anim Husb Vet Med. (2022) 11:68–71.

[32] Goodarzi Boroojeni F Senz M Kozłowski K Boros D Wisniewska M Rose D. The effects of fermentation and enzymatic treatment of pea on nutrient digestibility and growth performance of broilers. Animal. (2017) 11:1698–707. doi: 10.1017/S1751731117000787, PMID: 28416038

[33] Jazi V Boldaji F Dastar B Hashemi SR Ashayerizadeh A. Effects of fermented cottonseed meal on the growth performance, gastrointestinal microflora population and small intestinal morphology in broiler chickens. Br Poult Sci. (2017) 58:402–8. doi: 10.1080/00071668.2017.1315051, PMID: 28398088

[34] Cheng YH Hsiao FSH Wen CM Wu CY Dybus A Yu YH. Mixed fermentation of soybean meal by protease and probiotics and its effects on the growth performance and immune response in broilers. J Appl Anim Res. (2019) 47:339–48. doi: 10.1080/09712119.2019.1637344

[35] Akpoilih BU Adeshina I Chukwudi CF Abdel-Tawwab M. Evaluating the inclusion of phytase sources to phosphorus-free diets for GIFT tilapia (*Oreochromis niloticus*): growth performance, intestinal morphometry, immune-antioxidant responses, and phosphorus utilization. Anim Feed Sci Technol. (2023) 303:115678. doi: 10.1016/j.anifeedsci.2023.115678

[36] Tran N Shikuku KM Rossignoli CM Barman BK Cheong KC Ali MS. Growth, yield and profitability of genetically improved farmed tilapia (GIFT) and non-GIFT strains in Bangladesh. Aquaculture. (2021) 536:736486. doi: 10.1016/j.aquaculture.2021.736486

[37] Li X Zhang X Yao W Leng X. Dietary effects of cottonseed protein concentrate substituting fishmeal on the growth and flesh quality of Pacific white shrimp (*Litopenaeus vannamei*). Aquac Rep. (2023) 29:101482. doi: 10.1016/j.aqrep.2023.101482

[38] Liu Y Zhang Y Fan J Zhou H Huang H Cao Y. Effects of different viscous guar gums on growth, apparent nutrient digestibility, intestinal development and morphology in juvenile largemouth bass, *Micropterus salmoides*. Front Physiol. (2022) 13:927819. doi: 10.3389/fphys.2022.927819, PMID: 35991192 PMC9388778

[39] AOAC. Official methods of analysis of AOAC international, Association of Official Analysis Chemists International *18th ed*. (2005).

[40] Bian Y. Study on partial replacement of fish meal with fermented silkworm pupae meal in feeding largemouth bass, Micropterus salmoides. [master’s thesis]. Jiangsu, China: Jiangsu University of Science and Technology (2021).

[41] Zhang Y Zhou H Liu Y Zhu L Fan J Huang H. Dietary histamine impairs the digestive physiology function and muscle quality of hybrid grouper (*Epinephelus fuscoguttatus*♀ × *Epinephelus lanceolatus*♂). Antioxidants. (2023) 12:502. doi: 10.3390/antiox12020502, PMID: 36830060 PMC9952090

[42] Lin S Zhou X Zhou Y Kuang W Chen Y Luo L. Intestinal morphology, immunity and microbiota response to dietary fibers in largemouth bass, *Micropterus salmoide*. Fish Shellfish Immunol. (2020) 103:135–42. doi: 10.1016/j.fsi.2020.04.070, PMID: 32423866

[43] Liu Y Fu X Huang H Fan J Zhou H Deng J. High dietary histamine induces digestive tract oxidative damage in juvenile striped catfish (*Pangasianodon hypophthalmus*). Antioxidants. (2022) 11:2276. doi: 10.3390/antiox11112276, PMID: 36421462 PMC9686954

[44] Ren S Cai C Cui G Ni Q Jiang R Su X. High dosages of pectin and cellulose cause different degrees of damage to the livers and intestines of *Pelteobagrus fulvidraco*. Aquaculture. (2020) 514:734445. doi: 10.1016/j.aquaculture.2019.734445

[45] Cai C Ren S Cui G Ni Q Li X Meng Y. Short-term stress due to dietary pectin induces cholestasis, and chronic stress induces hepatic steatosis and fibrosis in yellow catfish, *Pelteobagrus fulvidraco*. Aquaculture. (2020) 516:734607. doi: 10.1016/j.aquaculture.2019.734607

[46] Fei S Liu H Han D Jin J Yang Y Zhu X. Apparent digestibility coefficients of several feed ingredients and their effect on digstive enzyme activies of juvenile yellow catfish (*pelteobagrus fulvidraco*). Acta hydrobiologica sinica. (2020) 44:1000–3207. doi: 10.7541/2020.043

[47] Pan J Han Y Huo P Su P Jiang Z. Dietary alginate oligosaccharide on intestinal morphology, activities of digestive enzymes and apparent digestibility of turbot (*Scophthalmus maximus* L). J Guangdong Ocean Univ. (2016) 36:39–44. doi: 10.3969/j.issn.1673-9159.2016.03.007

[48] Li J Wang C Wang L Zhao Z Luo L Xu Q. Effects of glutamate supplementation in low phosphorus diets on intestinal digestive enzyme activities and intestinal morphology of juvenile songpu mirror carp (*Cyprinus carpio L*.). J Guangdong Ocean Univ. (2019) 39:20–6. doi: 10.3969/j.issn.1673-9159.2019.04.004

[49] Vahouny GV Cassidy MM. Dietary fibers and absorption of nutrients. Proc Soc Exp Biol Med. (1985) 180:432–46. doi: 10.3181/00379727-180-42200 3001741

[50] Sinha AK Kumar V Makkar HPS De Boeck G Becker K. Non-starch polysaccharides and their role in fish nutrition—a review. Food Chem. (2011) 127:1409–26. doi: 10.1016/j.foodchem.2011.02.042

[51] Tonheim SK Nordgreen A Høgøy I Hamre K Rønnestad I. In vitro digestibility of water-soluble and water-insoluble protein fractions of some common fish larval feeds and feed ingredients. Aquaculture. (2007) 262:426–35. doi: 10.1016/j.aquaculture.2006.10.030

[52] Che M Yao C Lu Z Chi S Tan B. Effects of different amylose/amylopectin on diet processing characteristics, nutrient apparent digestibility and digestive enzyme activities of largemouth bass (*Micropterus salmoides*). Chin J anim nutr. (2022) 34:3220–32.

[53] Eusebio PS Coloso RM Mamauag REP. Apparent digestibility of selected ingredients in diets for juvenile grouper, *Epinephelus coioides* (Hamilton). Aquac Res. (2004) 35:1261–9. doi: 10.1111/j.1365-2109.2004.01148.x

[54] Li Y Deng J Tao L Yang X Bi B Pan J. Comparative study on apparent digestibility of five feed ingredients for juvenile GIFT strain of Nile Tilapia (*Oreochromis niloticus*). Feed Ind Mag. (2017) 38:22–7. doi: 10.13302/j.cnki.fi.2017.22.005

[55] Hartviksen M Vecino JLG Ringø E Bakke A-M Wadsworth S Krogdahl A. Alternative dietary protein sources for Atlantic salmon (*Salmo salar L*) effect on intestinal microbiota, intestinal and liver histology and growth. Aquac Nutr. (2014) 20:381–98. doi: 10.1111/anu.12087

[56] Caspary W. Physiology and pathophysiology of intestinal absorption. Am J Clin Nutr. (1992) 55:299S–308S. doi: 10.1093/ajcn/55.1.299s 1728844

[57] Li J Xu Q Wang C Luo L Zhao Z. Effects of glutamine and its precursors on intestinal digestive enzyme activity and intestinal morphology of songpu mirror carp (*Cyprinus carpio specularis*). Chin J Anim Nutr. (2014) 26:1347–52. doi: 10.3969/j.issn.1006-267x.2014.05.028

[58] Liu Y Zhou H Fan J Huang H Deng J Tan B. Assessing effects of guar gum viscosity on the growth, intestinal flora, and intestinal health of *Micropterus salmoides*. Int J Biol Macromol. (2022) 222:1037–47. doi: 10.1016/j.ijbiomac.2022.09.220, PMID: 36181882

[59] Wang K Wu L Dou C Guan X Wu H Liu H. Research advance in intestinal mucosal barrier and pathogenesis of crohn’s disease. Gastroenterol Res Pract. (2016) 2016:1–6. doi: 10.1155/2016/9686238, PMID: 27651792 PMC5019909

[60] Zhang H Zheng Y Zha X Ma Y Liu X Elsabagh M. Dietary l-arginine or n-carbamylglutamate alleviates colonic barrier injury, oxidative stress, and inflammation by modulation of intestinal microbiota in intrauterine growth-retarded suckling lambs. Antioxidants. (2022) 11:2251. doi: 10.3390/antiox11112251, PMID: 36421439 PMC9687183

[61] Vella A Farrugia G. D-lactic acidosis: pathologic consequence of saprophytism. Mayo Clin Proc. (1998) 73:451–6. doi: 10.1016/S0025-6196(11)63729-4, PMID: 9581587

[62] Gisbert E Andree KB Quintela JC Calduch-Giner JA Ipharraguerre IR Pérez-Sánchez J. Olive oil bioactive compounds increase body weight, and improve gut health and integrity in gilthead sea bream (*Sparus aurata*). Br J Nutr. (2017) 117:351–63. doi: 10.1017/S0007114517000228 28245885

[63] Liu Y Huang H Fan J Zhou H Zhang Y Cao Y. Effects of dietary non-starch polysaccharides level on the growth, intestinal flora and intestinal health of juvenile largemouth bass *Micropterus salmoides*. Aquaculture. (2022) 557:738343. doi: 10.1016/j.aquaculture.2022.738343

[64] Runkle EA Mu D. Tight junction proteins: from barrier to tumorigenesis. Cancer Lett. (2013) 337:41–8. doi: 10.1016/j.canlet.2013.05.038, PMID: 23743355 PMC3752309

[65] Chien SY Tsai CH Liu SC Huang CC Lin TH Yang YZ. Noggin inhibits *il-1β* and *bmp-2* expression, and attenuates cartilage degeneration and subchondral bone destruction in experimental osteoarthritis. Cell. (2020) 9:927. doi: 10.3390/cells9040927, PMID: 32290085 PMC7226847

[66] Zhang L Chen X Zheng P Luo Y Lu G Liu Z. Oral Bifidobacterium modulates intestinal immune inflammation in mice with food allergy. J Gastroenterol Hepatol. (2010) 25:928–34. doi: 10.1111/j.1440-1746.2009.06193.x 20546446

[67] Johnson IR Ball RO Baracos VE Field CJ. Glutamine supplementation influences immune development in the newly weaned piglet. Dev Comp Immunol. (2006) 30:1191–202. doi: 10.1016/j.dci.2006.03.003, PMID: 16697041

[68] Tian X. Effects of Bacillus aerobic fermented soybean meal instead of fish meal on growth performance, intestinal health and immune ability of largemouth bass (*Micropterus salmoides*). [Shanghai, China]. Shanghai Ocean University (2023) 38–39.

[69] Chen X Yi H Liu S Zhang Y Su Y Liu X. Promotion of pellet-feed feeding in mandarin fish (*Siniperca chuatsi*) by *Bdellovibrio bacteriovorus* is influenced by immune and intestinal flora. Aquaculture. (2021) 542:736864. doi: 10.1016/j.aquaculture.2021.736864

[70] Yu Z Sun Z Ou B Zhou M Huang Y Tan X. Effects of partial replacement of fish meal with black soldier fly (*Hermetia illucens*) larvae meal on growth performance, lipid metabolism and hepatointestinal health of juvenile golden pompano (*Trachinotus ovatus*). Aquac Rep. (2023) 33:101824. doi: 10.1016/j.aqrep.2023.101824

[71] Chen J Li Q Tan C Xie L Yang X Zhang Q. Effects of enrofloxacin’s exposure on the gut microbiota of Tilapia fish (*Oreochromis niloticus*). Comp Biochem Physiol Part D Genomics Proteomics. (2023) 46:101077. doi: 10.1016/j.cbd.2023.101077, PMID: 37080057

[72] Li L Song J Peng C Yang Z Wang L Lin J. Co-occurrence network of microbes linking growth and immunity parameters with the gut microbiota in Nile tilapia (*Oreochromis niloticus*) after feeding with fermented soybean meal. Aquac Rep. (2022) 26:101280. doi: 10.1016/j.aqrep.2022.101280

[73] Zhou L Zhong L Zhang S Chen X Liu H Wang M. Effects of fermented feed feeding mode on intestinal flora and metabolomics of channel catfish (*Ictalurus punctatus*). Prog Fish Sci. (2023) 44:1–10. doi: 10.19663/j.issn2095-9869.20230220001

[74] Zha L. The relationship between the recurrence of colorectal adenoma and the composition of intestinal flora. [doctoral’s thesis]. Shanghai, China: Shanghai Jiao Tong University (2017).

[75] Koh A De Vadder F Kovatcheva-Datchary P Bäckhed F. From dietary fiber to host physiology: short-chain fatty acids as key bacterial metabolites. Cell. (2016) 165:1332–45. doi: 10.1016/j.cell.2016.05.041, PMID: 27259147

[76] Semova I Carten JD Stombaugh J Mackey LC Knight R Farber SA. Microbiota regulate intestinal absorption and metabolism of fatty acids in the zebrafish. Cell Host Microbe. (2012) 12:277–88. doi: 10.1016/j.chom.2012.08.003, PMID: 22980325 PMC3517662

[77] Foysal MJ Nguyen TTT Chaklader MR Siddik MAB Tay C-Y Fotedar R. Marked variations in gut microbiota and some innate immune responses of fresh water crayfish, marron (*Cherax cainii*, Austin 2002) fed dietary supplementation of *Clostridium butyricum*. PeerJ. (2019) 7:e7553. doi: 10.7717/peerj.7553, PMID: 31523510 PMC6716501

[78] Costantini L Molinari R Farinon B Merendino N. Impact of omega-3 fatty acids on the gut microbiota. Int J Mol Sci. (2017) 18:2645. doi: 10.3390/ijms18122645, PMID: 29215589 PMC5751248

[79] Islam ZF Cordero PRF Feng J Chen Y-J Bay SK Jirapanjawat T. Two Chloroflexi classes independently evolved the ability to persist on atmospheric hydrogen and carbon monoxide. ISME J. (2019) 13:1801–13. doi: 10.1038/s41396-019-0393-0, PMID: 30872805 PMC6776052

[80] Ley RE Bäckhed F Turnbaugh P Lozupone CA Knight RD Gordon JI. Obesity alters gut microbial ecology. P Natl A Sci. (2005) 102:11070–5. doi: 10.1073/pnas.0504978102, PMID: 16033867 PMC1176910

[81] Binda C Lopetuso LR Rizzatti G Gibiino G Cennamo V Gasbarrini A. Actinobacteria: a relevant minority for the maintenance of gut homeostasis. Digest Liver Dis. (2018) 50:421–8. doi: 10.1016/j.dld.2018.02.012, PMID: 29567414

[82] Lin M Zeng CX Jia XQ Zhai SW Li ZQ Ma Y. The composition and structure of the intestinal microflora of *Anguilla marmorata* at different growth rates: a deep sequencing study. J Appl Microbiol. (2019) 126:1340–52. doi: 10.1111/jam.14174, PMID: 30552838

[83] Tsuchiya C Sakata T Sugita H. Novel ecological niche of *Cetobacterium somerae*, an anaerobic bacterium in the intestinal tracts of freshwater fish. Lett Appl Microbiol. (2008) 46:43–8. doi: 10.1111/j.1472-765X.2007.02258.x, PMID: 17944860

[84] Samsudin AA Asmara A Jahromi MF Shokryazdan P. Influence of dietary palm kernel cake on growth performance, carcass composition, meat quality, volatile fatty acids, intestinal bacteria population and villi histology of Cherry Valley ducks. *Malaysian*. J Anim Sci. (2017) 20:105–20.

