# Supplementary material for: Effects of replacing wheat bran with palm kernel cake or fermented palm kernel cake on the growth performance, intestinal microbiota and intestinal health of tilapia (GIFT, Oreochromis niloticus)
Source: Front Nutr. 2024 Feb 21;11:1368251. doi: 10.3389/fnut.2024.1368251 (PMC10915778; doi:10.3389/fnut.2024.1368251)
Supplement: Supplementary file 1 [file Data_Sheet_1.docx]

Supplementary Material

1. **2.1 Experimental diets**

PKC and FPKC were provided by Tongwei Group (Tongwei Co., Ltd., Chengdu, China). The specific fermentation process, the preparation of bacterial and enzyme solution was carried out with strict reference to the method provided by vland biotech (Qingdao, China). Before preparing FPKC, One tonne of PKC was added to 400 L of water and 10 kg of brown sugar. Then, 50 L of bacterial solution (yeast, lactic acid bacteria and bacillus) and 1 L of enzyme solution (protease, non-starch polysaccharide and cellulase enzyme) were added, and the materials were mixed well. Next, the materials were placed into plastic bags and compacted and sealed. Finally, After 4 days, take a sample of the fermented feed and add the material to distilled water at a ratio of 1:5. When the pH of the solution measured below 5.0, the fermentation was terminated.

## Supplementary Figures


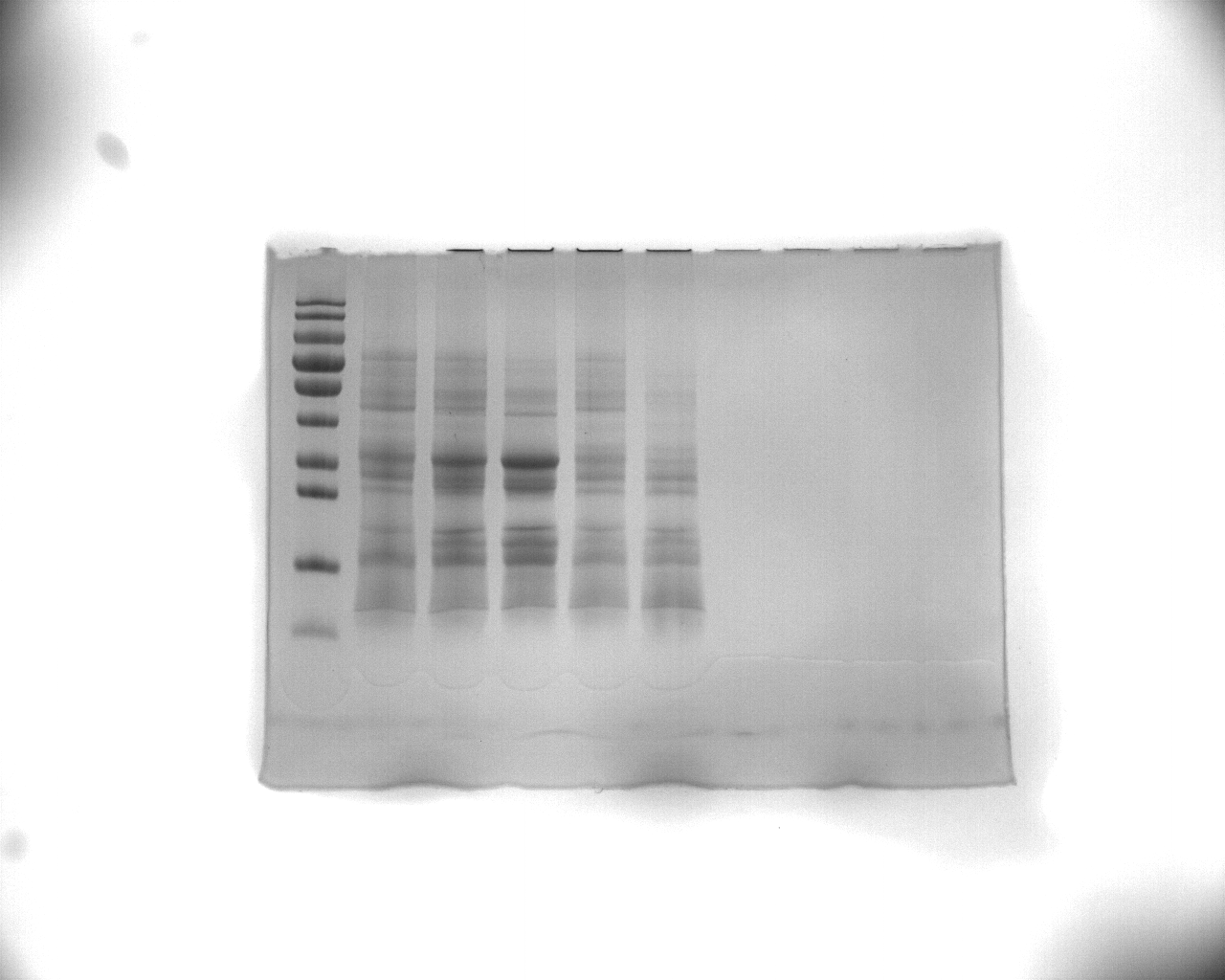


10

15

25

35

40

55

70

100

130

180

KDA

1 2 3 4 5 6

**Supplementary Figure 1.** The protein degradation of PKC and PKC by SDS-PAGE analysis.

1, Maker; 2, Control; 3, 40% PKC; 4, 100% PKC; 5, 40% FPKC; 6, 100% FPKC.
